# Necrotizing fasciitis with toxic shock syndrome in a child: a case report and review of literature

**DOI:** 10.1186/1757-1626-1-228

**Published:** 2008-10-08

**Authors:** Kotb Abass, Hekma Saad, Alaa A Abd-Elsayed

**Affiliations:** 1 Department of Paediatrics, Faculty of Medicine, Assiut University, Assiut, Egypt; 2 Department of Public Health and Community Medicine, Faculty of Medicine, Assiut University, Assiut, Egypt; 3 Department of Paediatrics, Al-Mabarah Hospital, Assiut, Egypt

## Abstract

**Introduction:**

Necrotizing fasciitis was described as early as the fifth century BC. It showed an increased incidence worldwide in the past several years.

**Case presentation:**

An 8-year-old Arabian boy was referred for admission as a case of cellulitis of the left thigh. Ten days prior to admission he had a cat scratch to his left thigh and the parents did not seek medical advice at that time. The child was again examined by orthopedic surgeon and a diagnosis of cellulites was made at that time.

Physical examination on admission revealed a very toxic appearing weak child with cold extremities and poor peripheral perfusion.

Examination of the left thigh revealed extensive swelling, induration and edema with dusky skin, blistering and bleb formation, in addition to an area of gangrenous skin. Laboratory investigation revealed white blood cell count of 22,400 × 10^9 ^with toxic granulation on peripheral blood smear.

The child was admitted to the pediatric intensive care unit and dopamine and dobutamine infusions were started after volume expansion. Penicillin and clindamycin also were started in addition to multiple transfusions of fresh frozen plasma. Surgical debridement of all necrotic tissues and drainage of involved fascia planes via extensive fasciotomy were done for our patient after stabilization of his vital signs and improvement of his general condition.

Blood cultures grew group A streptococcus, as did wound swab culture.

The child showed great improvements in his clinical condition after the 3^rd ^day of antibiotics and supportive treatment and the wound healed normally and antibiotics were administered for 21 days.

**Conclusion:**

Necrotizing fasciitis in children is a frequently misdiagnosed condition; early identification of the necrotizing process can improve the outcome of this life-threatening disease. Surgical debridement and antibiotics were the most important therapeutic measures.

## Introduction

Necrotizing fasciitis (NF) is a potentially fatal soft-tissue infection characterized by rapidly spreading inflammation and subsequent necrosis of the muscle fascia, subcutaneous fat and in some cases the epidermis [1]. NF was described as early as the fifth century BC [2]. An increasing incidence of NF caused by invasive Streptococcus pyogenes has been reported from various parts of world during the past several years [3]. In children, several features of NF differ from NF in adults. NF is common in immunocompromised and diabetic adult patients, while it frequently affects previously healthy children. Early surgical debridement and antibiotics are the most important therapeutic measures [4].

## Case presentation

An 8-year-old Arabian boy was referred for admission as a case of cellulitis of the left thigh. Ten days prior to admission he had a cat scratch to his left thigh and the parents did not seek medical advice at that time. Five days after the cat scratch he developed fever, swelling of his left thigh and limping of his left lower limb. At that time the child was seen by general practitioner and was started on oral Amoxicillin and Brufen (non steroidal anti-inflammatory drug) but due to non improvement, the child was again examined by orthopedic surgeon and a diagnosis of cellulites was made at that time. There was no history of trauma, injection or surgical operations, no history of recurrent infections or previous hospital admission.

Physical examination on admission revealed a very toxic appearing weak child with cold extremities and poor peripheral perfusion, Glasgow coma scale of 15/15, temperature of 39.8°C, pulse rate of 130/min., blood pressure of 80/50 mm and respiratory rate of 40/minute. There was no evidence of pallor, jaundice, lymphadenopathy and hepatosplenomegaly.

**Figure 1:**
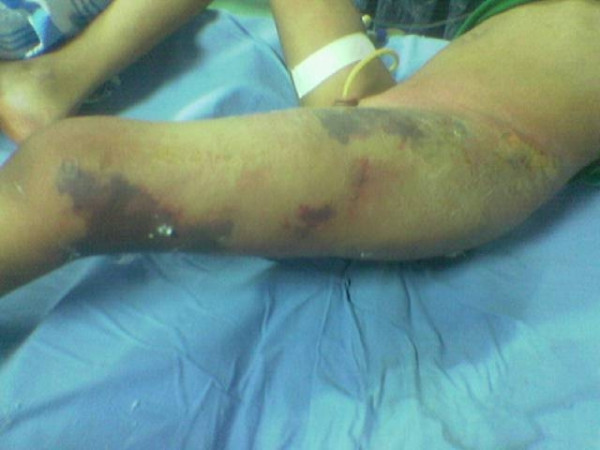
extensive swelling, induration and edema with dusky skin, blistering and bleb formation in addition to an area of gangrenous skin on examination of the left thigh.

Examination of the left thigh revealed extensive swelling, induration and edema with dusky skin, blistering and bleb formation, in addition to an area of gangrenous skin, figure 1. Laboratory investigations revealed white blood cell count of 22,400 × 10^9 ^(76% neutrophils, 4% lymphocytes, and 18% band forms) with toxic granulation on peripheral blood smear, the hemoglobin level was 9.3 g/dl, platelets count was 70 × 10^9^, ESR was 75 mm/h and CRP was 332 mg/ml, biochemical findings showed the following concentrations: urea 12 mmol/L, creatinine 124 umol/L, sodium 138 mmol/L, potassium 4 mmol/l, glucose 6 mmol/L, albumin 29 g/l, CPK level 1231 U/l, PT 19.5s (control: 15s), APTT 89s (Control 33s), AST 224 IU/l and AST 456 IU/l.

**Figure 2:**
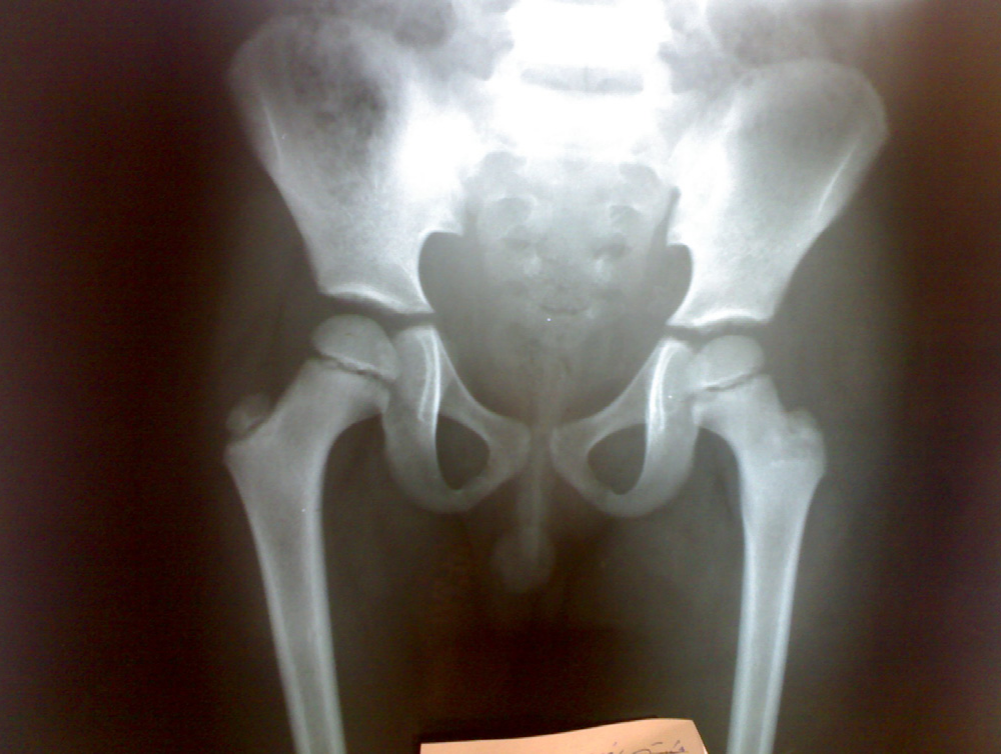
Plain x ray of both hips and thigh showed soft tissue edema of left thigh and normal left hip joint.

**Figure 3:**
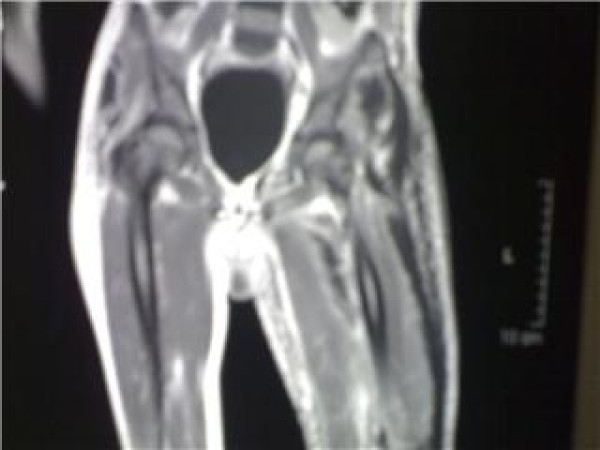
MRI of the left thigh revealed extension of inflammation along the fascial plains.

Plain x ray of both hips and thigh revealed soft tissue edema of left thigh and normal left hip joint, figure 2. Chest radiograph was normal. Doppler ultrasonographic examination of the left lower extremity revealed no abnormality. MRI of the left thigh revealed extension of inflammation along the fascial plains, figures 3.

**Figure 4:**
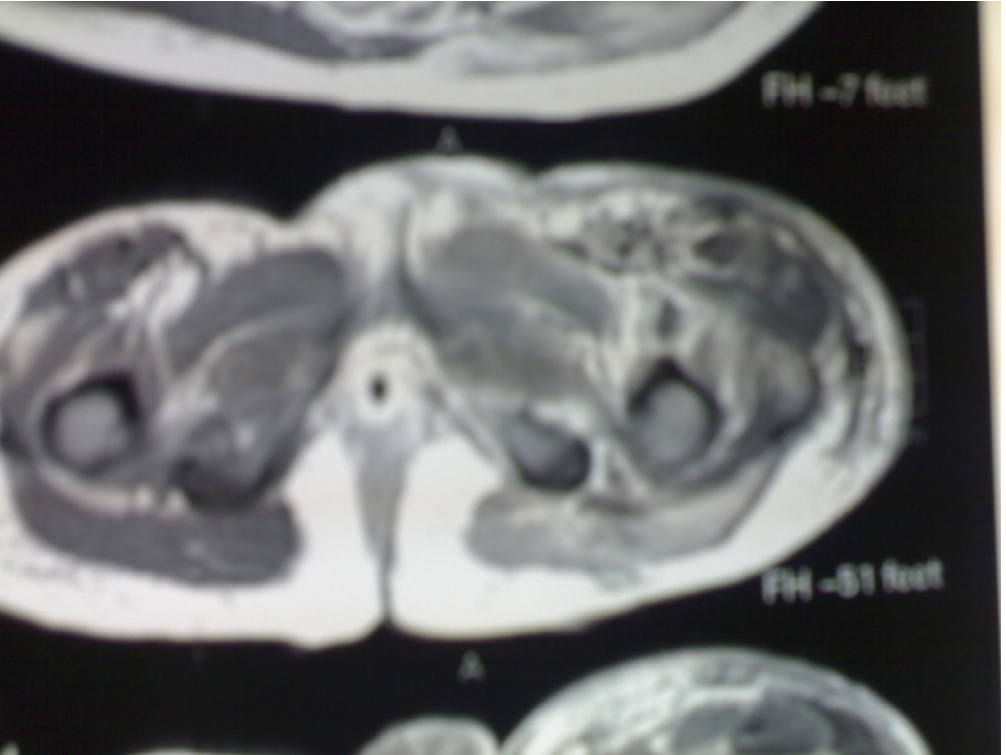
Surgical consultation was done and debridement of all necrotic tissues and drainage of involved fascia planes via extensive fasciotomy.

The child was admitted to the pediatric intensive care unit; dopamine and dobutamine infusions were started after volume expansion. Penicillin and clindamycin also were started in addition to multiple transfusions of fresh frozen plasma. Surgical consultation was requested; debridement of all necrotic tissues, figure 4, and drainage of involved fascia planes via extensive fasciotomy were performed for our patient after stabilization of his vital signs and improvement of his general condition.

Blood cultures grew group A streptococcus (GAS), as did wound swab culture, ASOT > 600 Todd units and histopathological examination confirmed the diagnosis of necrotizing fasciitis with myonecrosis.

The child showed great improvements in his clinical condition after the 3^rd ^day of antibiotics and supportive treatment. His biochemical abnormalities started to normalize, the wound healed normally and antibiotics were administered for 21 days.

## Discussion

NF is a rare condition in children [5]. It has been reported in 0.08 per100000 children per year with most lesions reported on the trunk [6].

NF begins with the introduction of bacterial infection, extensions of the bacterial infection along the fascial planes leading to necrosis of the superficial muscle fascia and deeper layers of the dermis, destruction and thrombosis of small blood vessel in the area leading to necrosis of the surrounding tissue. The extensive tissue damage often leads to systemic symptoms including multiorgan failure and shock [7]. There are two main categories of NF with different outcomes and therapeutic strategies. Type 1, which represents approximately 80 to 90% of all NF cases, caused by polymicrobial infection involving non- group A streptococci plus anaerobes and/or facultative anaerobes. Type 2, caused by monomicrobial infection by Group A streptococcus. Type 2 NF usually involves the extremities and limbs. These lesions can be complicated by septic shock known as streptococcal toxic shock syndrome (SST) characterized by hypotension and multiorgan failure [8].

Predisposing factors include trauma, surgery, burns and eczema, less commonly associated factors includes insect bites and subcutaneous insulin injection, an association between the use of non-steroidal anti-inflammatory and NF has been reported [9].

In our case report, skin break by cat scratch in addition to the use non-steroidal anti-inflammatory agents were the predisposing factors for NF. The use of non-steroidal anti-inflammatory agents may delay the diagnosis by attenuating the cardinal manifestations of inflammation. Furthermore, because Streptococcus pyogenes impair the phagocytic function and alter the host humoral immune responses, a minor infection with this agent may develop into a fulminant one [10].

In our patient, by the isolation of GAS from blood, tissue, hypotension with shock, renal and liver impairment, disseminated intravascular coagulopathy and necrotizing fasciitis, fulfillment of the diagnostic criteria of streptococcal TSS with NF was met as defined by the toxic shock case definition working group. The pathogenic mechanisms responsible for streptococcal TSS and NF, although not fully defined, it has been associated with streptococcal pyrogenic exotoxins [11].

The paucity of cutaneous findings early in the course of NF makes the diagnosis of this condition very difficult; NF in children is frequently misdiagnosed as simple soft tissue infections such as cellulitis, and this result in delay of the treatment [12]. Presence of vesiculation, ecchymosis, crepitus, anesthesia and necrosis are indicative of advanced disease. It is therefore important to diagnose NF before skin necrosis develops [13]. Delay in diagnosis was present in our case because the infection was confused with cellulitis. Initially, it is often difficult to differentiate cellulitis from NF. Clues that suggest NF rather than cellulitis include severe pain out of proportion to the skin findings which is not always easy to assess in children, rapidly spreading edema, bullae formation, mental status changes, marked leukocytosis and elevated creatinine kinase level [6]. Anesthesia of overlying skin can provide a clue that the process is NF rather than a simple cellulitis, because local skin anesthesia may antedate the appearance of skin necrosis [13]. Unless appropriate intervention is taken, there is likely to be a rapid evolution to cutaneous gangrene with myonecrosis and extension of inflammatory process. There are marked systemic symptoms, which may include shock and organ failure [3]. The early onset of shock, organ failure and the isolation of group A Streptococcus from a normally sterile site are the defining characteristics of the streptococcal TSS [3].

Magnetic resonance imaging (MRI) has the highest sensitivity (93–100%) for diagnosing NF. NF exhibits high signal intensity on T2-weighted images by MRI with hyperintense signal corresponding to fluids associated with NF. Using MRI, Rahmouni *et al*. were able to identify necrotizing soft-tissue infections which warrant immediate surgical intervention from non-necrotizing cellulitis, which can be treated medically [14].

Treatment of streptococcal NF includes include combination therapy of penicillin, clindamycin, prompt and aggressive exploration and debridement of suspected deep seated infection, and supportive measures for the management of shock and multiorgan failure [4]. The delay in antibiotic and surgical treatment probably affected the outcomes. I.V. immunoglobulin has been shown to reduce mortality if the necrotizing fasciitis is associated with TSS [15].

## Conclusion

NF in children is frequently misdiagnosed; early identification of the necrotizing process can improve the outcomes of this life-threatening disease. Surgical debridement and antibiotics were the most important therapeutic measures.

## List of abbreviations

NF: Necrotizing fasciitis; SST: Streptococcal toxic shock syndrome; GAS: Group A streptococcus; MRI: Magnetic resonance imaging

## Competing interests

The authors declare that they have no competing interests.

## Authors' contributions

KA & HS carried out the patient diagnosis, investigation, follow up and management. AAA-E carried out general coordination, drafting of the manuscript, writing the final manuscript and provided important suggestions. All authors read and approved the final manuscript.

## Consent

Written informed consent was obtained from the parents of our patient for publication of this case report and the accompanying images.
